# Clinical and Sociodemographic Characterization of Mexican Cohort with Pseudoarthrosis: A Retrospective, Cross-Sectional, and Descriptive Study

**DOI:** 10.3390/reports8040227

**Published:** 2025-11-05

**Authors:** Emilio Ignacio Pérez Jiménez, Félix Gustavo Mora Ríos, Brian Misael Muñoz Hernández, Josué Ramos Texta, Roberto Carlos Domínguez González, Joan Artemio Pérez Figueroa, Pedro García-Benavides, Carlos Alberto Castro-Fuentes

**Affiliations:** 1 Facultad Mexicana de Medicina, La Salle University, Las Fuentes 17, Tlalpan Centro I, Tlalpan, Mexico City 14000, Mexico; drmoraortoped@hotmail.com (F.G.M.R.); doctormisael@hotmail.com (B.M.M.H.); josue.rt2410@gmail.com (J.R.T.); dr.dominguez.222@gmail.com (R.C.D.G.); dr_joan_artemio@hotmail.com (J.A.P.F.); pedro1525@live.com (P.G.-B.); 2 Hospital Regional “General Ignacio Zaragoza” del ISSSTE, Calzada Ignacio Zaragoza 1711, Ejército, Constitucionalista I II y III, Iztapalapa, Mexico City 09220, Mexico; 3 Unidad de Investigación, Hospital Regional de Alta Especialidad de Ixtapaluca, Servicios de Salud del, Instituto Mexicano de Seguro Social Para el Bienestar (IMSS-BIENESTAR), Carretera Federal México-Puebla, Km 34.5, Ixtapaluca 56530, Mexico

**Keywords:** clinical, sociodemographic, characterization, pseudoarthrosis, Mexican cohort

## Abstract

Background/Objectives: Pseudoarthrosis continues to be a public health problem; however, in our country, information is scarce, particularly when talking about the clinical and sociodemographic characterization of the Mexican population with pseudoarthrosis. Methods: In this study, clinical and sociodemographic characteristics such as sex, age, educational level, nutritional status, comorbidities, affected bone, fracture characteristics, degree of exposure, and waiting time for the patient to undergo surgery were analyzed. Results: A total of 267 patients were included in the present study. A higher frequency of men (53.6%) was identified compared to women, and the main age group was 46–75 years (50.1%). The main comorbidities identified in our population were smoking (*n* = 141; 52.8%) and osteoporosis (*n* = 84; 31.5%). When evaluating the clinical characteristics of pseudoarthrosis, the tibia (*n* = 65; 24.3%) and radius (*n* = 54; 20.2%) were the main bones affected. Fracture exposure could be identified in 17.65% (*n* = 47) of the population, and the main grade of involvement was II (46.8%). Regarding the prevalence of the affected bone, the tibia was the main one, with 13.5% (9.38–17.58%) female patients, 12.7% (8.73–16.73%) with overweight, 18.0% (13.37–22.59%) with osteoporosis, and 14.2% (10.04–18.42%) with a history of smoking. Conclusions: In our cohort, we identified a high prevalence of the tibia as the affected bone, while overweight, obesity, osteoporosis, and smoking were the clinical and sociodemographic characteristics that characterized our population. The findings of this study lay the groundwork for understanding the clinical and sociodemographic context of a Mexican cohort with nonunion.

## 1. Introduction

Pseudoarthrosis is a total disability in which a fracture cannot perform the consolidation process, due to a failure in osteogenesis, with an estimated time of nonunion occurring at six or eight months (nonunion), depending on the authors [1].

According to previous studies, pseudoarthrosis complicates fractures in 1–5% of cases, particularly fractures of long bones, such as the humerus, femur, tibia, and radius in 10% of cases, while, when external factors such as inadequate fixation or approximate control of rotation intervene in diaphyseal fractures of long bones, the incidence of pseudoarthrosis in these cases is 50% [2].

The main factors that condition the appearance of pseudoarthrosis have been identified as sex, smoking, age, race, nutrition, diabetes mellitus, infection, and obesity [3,4,5]. In addition, decalcifying osteopathy, as well as the type of fracture, are factors related to treatment, as insufficient reduction can condition this pathology [6,7,8]. However, in Mexico, the clinical and sociodemographic context in which pseudoarthrosis occurs is still unknown.

The therapeutic management carried out to date has shown that surgical intervention is the mainstay of treatment, and in necessary cases between two and five surgeries are needed. However, it is also the result of spinal deformity surgeries in adults and children, such as pediatric cervical spine fusions [9,10]. Furthermore, the osteosynthesis material used in the treatment of pseudoarthrosis will influence the patient’s outcome. In this regard, the use of external fixators, nails, and plates, in addition to conductive osteosynthesis, primarily determines the patient’s outcome [11,12,13]. Meanwhile, the use of certain materials such as closed-cell interbody spacers for posterior lumbar interbody fusion (PLIF) can cause pseudoarthrosis in elderly patients [14].

Despite being a common condition, information regarding the factors that determine its etiopathogenesis is scarce, and the predominant clinical and sociodemographic characteristics of the Mexican population with nonunion have not yet been clearly established. Therefore, the objective of this study is to provide an initial exploration of the clinical and sociodemographic characteristics of a Mexican cohort with nonunion from a single institution.

## 2. Materials and Methods

### 2.1. Patients

This is a retrospective, cross-sectional, and observational study with 267 patients treated by the traumatology and orthopedics service of the “General Ignacio Zaragoza” Regional Hospital (HRGIZ) of the Institute of Security and Social Services of State Workers (ISSSTE) during the period from January 2011 to January 2015. It was approved by the Hospital Ethics Committee (535–2024), and the included patients met the following selection criteria:

### 2.2. Inclusion Criteria

- Patients with a clinical and/or radiological diagnosis of pseudoarthrosis.
- Patients treated at the “General Ignacio Zaragoza” Regional Hospital of the ISSSTE.
- Patients who agreed to and signed the consent form to participate in the study.

### 2.3. Exclusion Criteria

- Patients with bone infections caused by microbial agents.
  - Patients with incomplete medical records.
  - Patients with unstable fractures.

**Figure 1:**
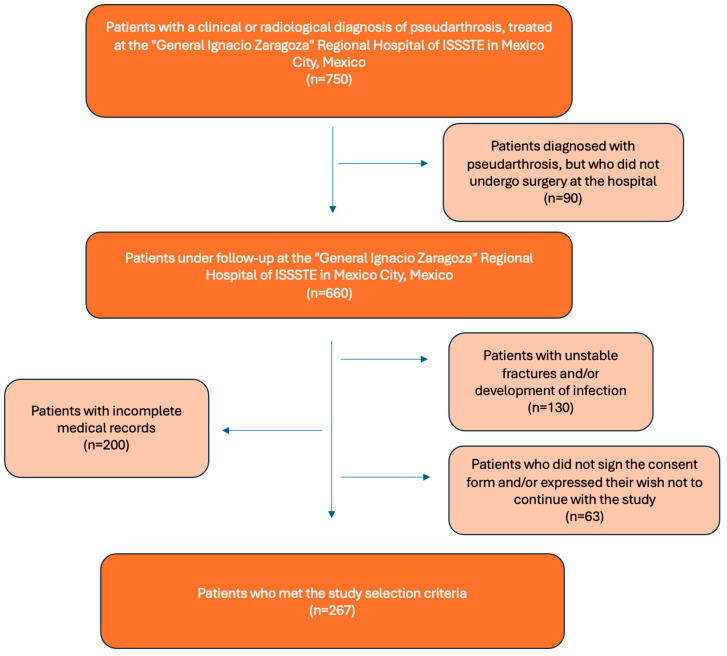
Flowchart showing the process of selecting patients for the present study, based on the selection criteria.

Figure 1 shows the flowchart illustrating the population screening process used to identify patients who met the study selection criteria.

### 2.4. Pseudoarthrosis

In this study, pseudoarthrosis was defined as a serious outcome of nonunion of a fracture that fails to heal within the expected timeframe, characterized by persistent pain and disability. In this condition, a fibrous or cartilaginous space forms at the fracture site, mimicking a false joint; this condition is the result of biological, mechanical, and systemic factors that interfere with the healing process [15].

The diagnosis of pseudoarthrosis was made based on clinical and imaging diagnosis, according to Milano and González [16]. In addition, the affected bone (humerus, femur, tibia, fibula, radius, ulna, and others) was determined. Likewise, the frequency and grade of exposure of the fracture (I, II, IIIa, IIIb, or IIIc) presented by each patient included in the present study was identified, according to the classification of Gustillo [17]:

Grade I: Low energy; clean wound < 10 mm.

Grade II: Moderate energy; clean/minimally contaminated wound > 10 mm.

Grade IIIa: High energy; good skin coverage, contaminated.

Grade IIIb: High energy; extensive soft tissue injury, massive contamination, bare bone.

Grade IIIc: Vascular injury, requires repair.

### 2.5. Operational Definition of Overweight and Obesity

According to the most recent publication of the World Health Organization [18], and for the operational purposes of this work, overweight and obesity in the adult population were defined as follows:

Overweight: A condition characterized by excessive fat accumulation resulting from an imbalance between caloric intake (diet) and caloric expenditure (physical activity). This condition is determined by a body mass index (BMI) equal to or greater than 25 kg/m^2^.

Obesity: A chronic disease resulting from excessive fat accumulation that puts the health of the person who suffers from it at risk. Obesity can lead to an increased risk of type 2 diabetes and heart disease, can affect bone health and reproduction, and increases the risk of developing some types of cancer. This condition, like overweight, is determined by the BMI when it is equal to or greater than 30 kg/m^2^.

### 2.6. Statistical Analysis

A database was constructed for the population that comprised this study and that met the selection criteria. The variables considered were sex, age, education, nutritional status, comorbidities (hypertension, osteoporosis, diabetes mellitus, alcoholism, smoking, and other substances), bone affected by pseudoarthrosis, fracture characteristics, exposure and degree of exposure, and, finally, waiting time for surgery [19,20,21]. These variables were analyzed and expressed as frequencies and percentages. In addition, the prevalence of clinical and sociodemographic characteristics associated with the bone affected by the nonunion was determined using the normal approximation formula. This was performed by calculating the ratio of the number of patients with the clinical characteristic to the sample size. The standard error was then determined to calculate the confidence interval for the prevalence. From this, the upper and lower limits of the 95% confidence interval were determined. All statistical analyses were performed using Jamovi (version 2.6.2).

## 3. Results

A total of 267 patients were included in the present study, of which 53.6% (*n* = 143) were men and the rest were women (Table 1). The main age group was 46–75 years (*n* = 134; 50.1%), followed by 19–45 (*n* = 83; 31.0%), >76 (*n* = 28; 10.48%), and 4–18 (*n* = 22; 8.2%).

Regarding the schooling of our population, 36.7% (*n* = 98) had a bachelor’s degree. On the other hand, 42.7% (*n* = 114) were overweight and 33.7% (*n* = 90) were obese. Among the comorbidities identified, the most common were smoking 52.8% (*n* = 141) and osteoporosis 31.5% (*n* = 84).

When evaluating the clinical characteristics of pseudoarthrosis in the population, among the affected bones, the tibia was identified as being first (*n* = 65; 24.3%), followed by the radius (*n* = 54; 20.2%), the femur (*n* = 53; 19.9%), the humerus (*n* = 53; 19.9%), others (*n* = 17; 6.4%), the fibula (*n* = 15; 5.6%), and the ulna (*n* = 10; 3.7%) (Table 2). On the other hand, the frequency of fractures exposed was identified in 17.65% (*n* = 47) of cases and the grades of fracture exposure identified were grade I, II, IIIa, and IIIb. Of these, grade II was the main grade identified, with a frequency of 46.8% (*n* = 22), followed by grade I (*n* = 13; 27.75%), grade IIIb (*n* = 10; 21.3%) and grade IIIa (*n* = 2; 4.3%). No grade IIIc fractures were identified. Finally, the surgical waiting time for more than 50% of the population was between 8 and 14 days.

When evaluating the affected bone in the study population, of the total number of patients, particularly in the case of the female sex, the tibia was the main affected bone (13.5%) (9.38–17.58%) (Table 3), while, in the population that presented obesity, the humerus (9.7%) (6.19–13.29%) was identified as the main affected bone, unlike the case of overweight patients, where the tibia (12.7%) (8.73–16.73%) was the main affected bone. The above is similar for patients diagnosed with osteoporosis (18.0%) (13.37–22.59%). In the case of patients with a history of SAH and DM, the main affected bone was the femur (6.7% (3.74–9.74%) and 8.6% (5.24–11.98%), respectively), while the humerus (4.9%) (2.28–7.465%) and tibia (4.9%) (2.28–7.46%) were the most affected bones in the population with a history of alcoholism. Finally, in the case of patients with a history of smoking, the tibia (14.2%) (10.04–18.42%), humerus (13.9%) (9.72–18.00%) and radius (10.1%) (6.50–13.72%) were the main bones affected.

## 4. Discussion

In our cohort, a higher frequency of men (53.6%) was identified compared to women, where the main age group affected was 46–75 years (50.1%). A total of 42.7% of the population was overweight, followed by obesity (33.7%). The main comorbidity identified in our population was smoking and osteoporosis. When evaluating the clinical characteristics of pseudoarthrosis, the tibia was the main bone affected (24.3%). Fracture exposure could be identified in 17.65% of the population, and the main grade of involvement was II (46.8%). Meanwhile, the time in which the patient underwent surgery was no longer than two weeks for 53.6% of the population studied.

In our cohort, a greater involvement of pseudoarthrosis was identified in male patients. This is similar to that which was reported by Chicaiza Calle et al. [6], with a mean age of 37.2 years, while in our study population the average age was 49.3 years. Particularly, the main age group affected was 46–75 years old (50.1%). However, studies such as that of Mongonza et al. [2] report completely different results, since in this study, 83.3% of patients with pseudoarthrosis in a hospital in the Congo were women, whereas the main age groups identified were 21–30 years (27.7%) and 61–70 years (22.2%). However, in a retrospective study in a Mexican cohort conducted by Mora Ríos et al. [11], pseudoarthrosis had a frequency of 2% in the study population. Of the total population, 71% were men, with an average age of 45 years, being very similar data to those we are reporting. The differences in the previous studies are probably due to the population proportionality and the professional activities performed by the patients included in the studies.

Among the comorbidities identified in this study, smoking (52.8%), osteoporosis (31.5%), and diabetes mellitus (26.6%) stand out, the same comorbidities as those reported in the study by Chicaiza Calle et al. [5], with a frequency of 21% for diabetes mellitus and 15% for systemic arterial hypertension. However, for other studies, such as Da Costa et al. [22], arterial hypertension has occupied the first position (42%). On the other hand, in the study by Chicaiza Calle et al. [6], it was observed that 24%, 13%, and 8% of patients with tibial pseudoarthrosis were chronic users of cigarettes, alcohol, and corticosteroids, respectively. Particularly, alcohol consumption and smoking were clinical conditions identified in our population at 14.6% and 52.8%, respectively.

Among the clinical characteristics of pseudoarthrosis, bone affectations such as tibia (24.3%), radius (20.2%), femur (19.9%), humerus (19.9%), fibula (5.6%), and ulna (3.7%) were identified. Regarding humeral shaft fractures, minimally invasive plate osteosynthesis has shown good results; however, the presence of complications such as pseudoarthrosis are identified. Mouraria et al. [23] conducted a retrospective study in patients with minimally invasive osteosynthesis, where 70.7% were men, while 9.3% developed pseudoarthrosis. This is consistent with what was reported by Mora Ríos et al. [11], where pseudoarthrosis was diagnosed in the tibia (47%), followed by the femur (26%), humerus (10%), and clavicle (5%), in such a way that it seems to be a clinical characteristic of high prevalence of pseudoarthrosis in our population. Despite the similarity of both studies carried out in the Mexican population, the frequency of pseudoarthrosis in the tibia and femur seems to be high according to several studies worldwide.

When identifying the types of fractures in our population, grade II fractures were mainly identified in almost 50% of the population. The findings differ from those reported by Da Costa et al. [22], where grade IIIa fractures were the most prevalent (41.93%), followed by grade II (36.77%) and grade I (15.84%) fractures. It is important to mention that, by not considering patients with an infectious process, a high number of patients with grade III fractures were eliminated from the population. Therefore, a high prevalence of grade III fractures was likely not identified in our population. Regarding grade III fractures, after grade IIIa fractures, grade IIIb (4.51%) and grade IIIc fractures (1.29%) were identified. In our cohort, grade IIIb fractures (21.3%) exceeded grade IIIa fractures (4.3%) and no grade IIIc fractures were identified.

Although in the present study it was not possible to identify the risk factors or to know their association with pseudoarthrosis, in studies conducted in other countries, the factors that demonstrated statistical significance were alcohol consumption, tobacco, and open fractures [2]. Additionally, Jensen et al. [3] identified that pseudoarthrosis was associated with smoking, open fractures, diabetes, infection, and obesity. In addition to the traditional risk factors, in cases such as isthmic spondylolisthesis treated with a pedicle screw system in posterolateral fusion, radiological risk factors for pseudoarthrosis have been reported. Particularly, the preoperative percentage disk height (odds ratio: 3.60 per 10%, *p* < 0.01) and slip angle (odds ratio: 4.48 per 10 degrees of kyphosis, *p* < 0.05) were the most crucial risk factors for nonunion [24].

The infectious factor is known to represent from 23.5 to 50% of the origin of pseudoarthrosis. Although this conditioning factor for the appearance of pseudoarthrosis was not considered in the present study, it is necessary to identify whether there is an infectious process at the bone or intraosseous level [1]. However, it appears to be one of the most prevalent risks in worldwide studies (Jensen et al. [3]). In this sense, some studies have reported that, in the case of spinal surgical interventions, they developed pseudoarthrosis associated with surgical site infection (5.0%), with the number of fused levels being the main predictor of the development of pseudoarthrosis (odds ratio [OR], 1.356/level, *p* < 0.001), where 85.7% *Staphylococcal* species were identified, of which 27.8% exhibited methicillin-resistant *Staphylococcus aureus* (*MRSA*) [25].

Although this study did not analyze the type of approach that was implemented in the cohort, the time that elapsed between the diagnosis of pseudoarthrosis and the time in which the surgical intervention took place was identified. It is noteworthy that more than 50% of the population was operated on between day 8 and day 14, followed by days 15–21 (32.2%), and only 14.2% (*n* = 38) was operated on between day 0 and day 7. Although in other series the intervention occurred on the same day of hospital admission, with a maximum time of >180 min [17], the surgical intervention was a single occurrence, which is consistent with what was reported by Mogonza et al. [2], while, in some cases, pseudoarthrosis is the result of surgery for spinal deformities in the adult population [26]. In this sense, pseudoarthrosis was associated with the length of the fusion (OR = 1.17, 95% CI = 1.05–1.292, *p* = 0.004), osteotomy (OR = 0.28, 95% CI = 0.09–0.85, *p* = 0.025), pelvic fixation (OR = 0.34, 95% CI = 0.13–0.88, *p* = 0.026), and combined approaches (OR = 3.29, 95% CI = 1.09–9.91, *p* = 0.034) [27], in such a way that it is highlighted that the anterior surgical approaches decrease the incidence of pseudoarthrosis by 30%. Additionally, it has been associated with complications of spinal surgery, such as durotomy. Bydon et al. [28] conducted a study in which they compared a group of patients with durotomy and a group without durotomy, during a 1-year follow-up. Pseudoarthrosis was significantly higher in the durotomy group (35.29%), compared to the group without durotomy. Patients with durotomy showed a 2.23 times higher risk of developing pseudoarthrosis (RR 2.23; 95% confidence interval 1.05–4.75) when compared to patients without durotomy.

Finally, early management and timely recognition of the clinical and sociodemographic characteristics that lead to pseudoarthrosis in patients will allow a timely approach and thus improve the patient’s quality of life [29]. In this regard, studies such as that of Pennington et al. [30] have demonstrated the impact of nonunion on patients with pseudoarthrosis, by comparing two groups with and without nonunion using the five-dimensional EuroQol questionnaire. A total of 64% of patients with nonunion had worse quality-adjusted life years scores compared to only 9% of controls (*p* < 0.01). Likewise, lower scores were identified in mental health (*p* < 0.01) and disability due to pain (*p* < 0.01). Consequently, an increase in direct and postoperative costs (*p* < 0.01) was identified.

This study presented some limitations, mainly due to its retrospective nature, whose inherent limitation is information bias. Due to its single-center design, the sample size was small. Finally, due to the absence of a control group, it was not possible to identify risk factors and their association with the development of nonunion. Therefore, the results obtained in this study do not fully represent the general Mexican population, and the findings should be interpreted with caution. However, to our knowledge, this is the first study to show the prevalence of clinical and sociodemographic characteristics in a Mexican cohort with nonunion.

## 5. Conclusions

In our cohort, a higher percentage of men than women were identified (53.6% vs. 46.4%). The main comorbidities identified in our population were smoking (*n* = 141; 52.8%) and osteoporosis (*n* = 84; 31.5%). Regarding pseudoarthrosis, the main bones affected were the tibia and radius, while the degree of fracture was type II or I.

Regarding the prevalence of the affected bone, the tibia was the main one with female patients, some with overweight, osteoporosis, or with a history of smoking.

The findings of this study establish the initial foundations for understanding the clinical and sociodemographic context of a Mexican cohort with pseudoarthrosis.

## Figures and Tables

**Table 1:** Clinical and sociodemographic characteristics of the study population.

Variable	Frequency (n)	Percentage
Sex		
Female	124	46.4%
Age (years)		
4–18	22	8.2%
19–45	83	31.0%
46–75	134	50.1%
>76	28	10.48%
Schooling		
Elementary	39	14.6%
Secondary school	39	14.6%
High school	88	33.0%
Bachelor’s degree	98	36.7%
Without schooling	3	1.12%
Nutritional status		
Overweight	114	42.7%
Obesity	90	33.7%
Comorbidity		
Osteoporosis	84	31.5%
Systemic arterial hypertension	59	22.1%
Diabetes mellitus	71	26.6%
Alcoholism	39	14.6%
Smoking	141	52.8%

**Table 2:** Clinical characteristics of Pseudoarthrosis.

Variable	Frequency (n)	Percentage
Affected bone		
Humerus	53	19.9%
Femur	53	19.9%
Tibia	65	24.3%
Fibula	15	5.6%
Radius	54	20.2%
Ulna	10	3.7%
Other	17	6.4%
Open fracture		
Yes	47	17.65%
No	220	82.4%
Grade of fracture exposure		
I	13	27.75
II	22	46.8%
IIIa	2	4.3%
IIIb	10	21.3%
Surgical lead time (days)		
0–7	38	14.2%
8–14	143	53.6%
15–21	86	32.2%

**Table 3:** Prevalence and confidence interval (95%) of clinical and sociodemographic characteristics with bone affected by pseudoarthrosis in the studied population.

	Females (95% CI)	Obesity (95% CI)	Overweight (95% CI)	Osteoporosis (95% CI)	SAH (95% CI)	DM (95% CI)	Alcoholism (95% CI)	Smoking (95% CI)	Open Fracture (95% CI)
Humerus	7.9% (4.64–11.10%)	9.7% (6.19–13.29%)	7.9% (4.64–11.10%)	2.2% (0.47–4.03%)	3.0% (0.96–5.04%)	4.5% (2.00–6.98%)	4.9% (2.28–7.46%)	13.9% (9.72–18.00%)	1.1% (0.00–2.37%)
Femur	9.0% (5.56–12.42%)	5.2% (2.57–7.91%)	7.9% (4.64–11.10%)	5.6% (2.86–8.38%)	6.7% (3.74–9.74%)	8.6% (5.24–11.98%)	1.5% (0.04–2.96%)	6.4% (3.45–9.29%)	4.1% (1.73–6.51%)
Tibia	13.5% (9.38–17.58%)	9.0% (5.56–12.42%)	12.7% (8.73–16.73%)	18.0% (13.37–22.59%)	6.0% (3.15–8.83%)	5.2% (2.57–7.91%)	4.9% (2.28–7.46%)	14.2% (10.04–18.42%)	7.1% (4.04–10.20%)
Fibula	3.4% (1.21–5.53%)	1.1% (0.00–2.37%)	3.4% (1.21–5.53%)	1.5% (0.04–2.96%)	0.4% (0.07–2.09%)	0.4% (0.07–2.09%)	0.7% (0.21–2.69%)	3.4% (1.21–5.53%)	0.7% (0.21–2.69%)
Radius	9.4% (5.87–12.85%)	5.6% (2.86–8.38%)	6.4% (3.45–9.29%)	3.0% (0.95–5.04%)	2.6% (0.71–4.54%)	5.2% (2.57–7.91%)	1.5% (0.04–2.96%)	10.1% (6.50–13.72%)	3.7% (1.48–6.02%)
Ulna	1.5% (0.04–2.96%)	1.1% (0.00–2.37%)	1.5% (0.04–2.96%)	0.0% (0.00–1.42%)	0.4% (0.07–2.09%)	0.4% (0.07–2.095)	0.7% (0.21–2.69%)	2.2% (0.47–4.03%)	0.7% (0.21–2.69%)
Another affected bone	1.9% (0.61–4.30%)	1.9% (0.61–4.30%)	3.0% (1.30–5.86%)	1.1% (0.23–3.25%)	3.0% (1.30–5.86%)	2.2% (0.83–4.81%)	0.4% (0.01–2.08%)	2.6% (1.06–5.29%)	0.0% (0–1.12%)

## Data Availability

The original contributions presented in this study are included in the article. Further inquiries can be directed to the corresponding author.
